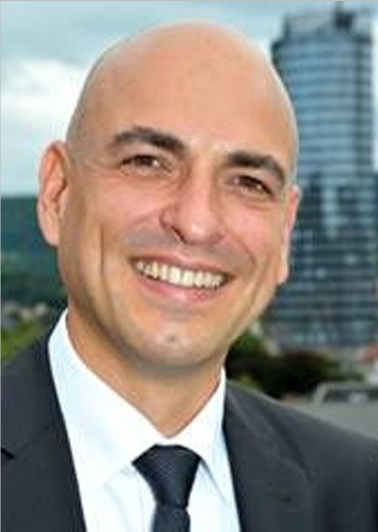# DNA Origami Meets Polymers: A Powerful Tool for the Design of Defined Nanostructures

**DOI:** 10.1002/anie.202005907

**Published:** 2020-10-28

**Authors:** Nadine Hannewald, Pia Winterwerber, Stefan Zechel, David Y. W. Ng, Martin D. Hager, Tanja Weil, Ulrich S. Schubert

**Affiliations:** ^1^ Laboratory of Organic and Macromolecular Chemistry (IOMC) Friedrich Schiller University Jena Humboldtstrasse 10 07743 Jena Germany; ^2^ Jena Center for Soft Matter (JCSM) Friedrich Schiller University Jena Philosophenweg 7 07743 Jena Germany; ^3^ Max Planck Institute for Polymer Research Ackermannweg 10 55128 Mainz Germany

**Keywords:** DNA origami, hybrid materials, nanostructures, polymer chemistry, surface modification

## Abstract

The combination of DNA origami nanostructures and polymers provides a new possibility to access defined structures in the 100 nm range. In general, DNA origami serves as a versatile template for the highly specific arrangement of polymer chains. Polymer‐DNA hybrid nanostructures can either be created by growing the polymer from the DNA template or by attaching preformed polymers to the DNA scaffold. These conjugations can be of a covalent nature or be based on base‐pair hybridization between respectively modified polymers and DNA origami. Furthermore, the negatively charged DNA backbone permits interaction with positively charged polyelectrolytes to form stable complexes. The combination of polymers with tuneable characteristics and DNA origami allows the creation of a new class of hybrid materials, which could offer exciting applications for controlled energy transfer, nanoscale organic circuits, or the templated synthesis of nanopatterned polymeric structures.

## Graphical abstract

The combination of DNA origami with synthetic polymers results in a new class of materials with interesting properties and characteristics. This approach is more precise than conventional nanofabrication methods, such as the positioning of single polymer strands. This Minireview shows the potential preparation of nanostructures from polymers and two‐ or three‐dimensional DNA origami.

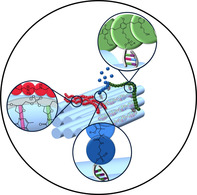

## 1 Introduction

The fabrication of functional nanoparticles and defined nanoscale surfaces represents an intensively investigated topic of current research. Besides the synthesis of such materials, the improvement in the fabrication of smaller and more precise geometries as well as the development of precisely addressable surfaces is also of interest. Significant improvements in such fabrication techniques could be of further usage for, for example, reducing the size of data storage, optical devices, or the development of new drug‐delivery systems.[^1^, ^2^, ^3^]

Such nanostructures can be fabricated in many ways, but two of the most important methods are lithography and self‐assembly. Lithography, as a top‐down technique, enables manipulation of larger objects to result in smaller‐size geometries with the desired shape.^[4]^ Nevertheless, it often requires expensive and complicated setups, thus making the fabricated samples expensive and not suitable for the large‐scale fabrication of nanostructures.^[5]^ In contrast, self‐assembly, as a bottom‐up process, relies on the interactions of the assembling units without any external stimuli, which will be discussed in the following only for small molecules.^[4]^ Such moieties can be, for example, based on hydrogen bonding,^[6]^ van der Waals forces,^[7]^ hydrophobic and hydrophilic[^8^, ^9^] interactions, or π‐π stacking.^[10]^ During the self‐assembly processes of synthetic molecules, various desired structures could be formed, which makes this process a low cost and fast alternative compared to lithography.^[11]^ However, not all geometries can be realized in this way.

**Figure 1:**
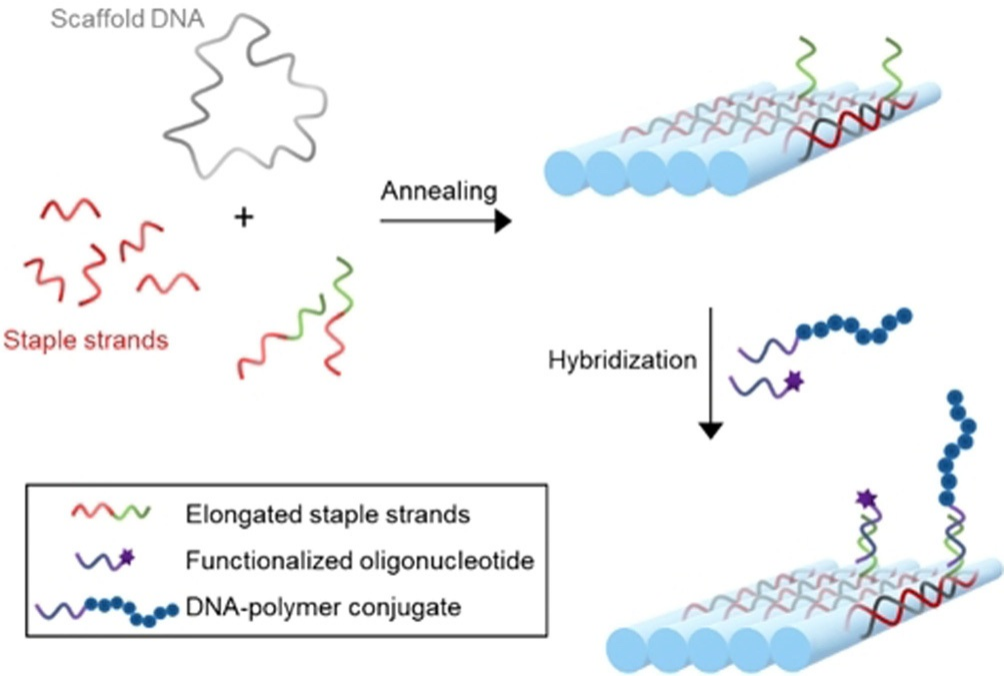
DNA origami structures are created by annealing a scaffold DNA, respectively designed staple strands, and elongated staple strands. The protruding DNA strands can be hybridized to complementary, functionalized oligonucleotides and DNA‐polymer conjugates.

One prominent example for a versatile self‐assembling process in nature is the formation of the DNA double helix, which is based on hydrogen bonding between complementary base pairs. In 1982, Seeman took inspiration from such processes and realized the folding of DNA into designed superstructures.[^12^, ^13^] This idea was further expanded by Rothemund in 2006, by establishing the so‐called DNA origami technology, which led to a breakthrough in the construction of DNA objects.^[14]^ In this approach, a long, circular single‐stranded DNA (“scaffold DNA”) is folded into a distinct shape with the help of a set of short “staple strands”. These staple strands are designed to hybridize to complementary sequences within the scaffold DNA. Elongating particular staple strands by short oligonucleotides results in surface‐ protruding single‐stranded DNA (ssDNA), which can subsequently undergo hybridization to additional molecules. (Figure 1) Thus, DNA origami provides a precisely addressable surface and has been shown to be a powerful tool for the distinct positioning of, for example, nanoparticles in a predefined manner.[^12^, ^15^]

In this Minireview, we focus on the functionalization of DNA origami nanostructures with synthetic polymers or polymer‐oligonucleotide conjugates to afford unique hybrid nanostructures that are very challenging to achieve with other techniques. However, in contrast to previous contributions, the pure self‐assembly behavior of polymer‐DNA conjugates will not be discussed.[^16^, ^17^, ^18^] However, a range of methods for the attachment of polymers onto DNA nanostructures in a predesigned manner is described in detail. Additionally, the advancements as well as the limitations of the functionalization of DNA origami is discussed and compared to lithography and traditional self‐assembly methods. Finally, we show that the functionalization of DNA origami can be a powerful tool for the preparation of polymeric nanostructured objects.

## 2 Polymers and DNA Origami: How to Bridge the Fields

**Figure 2:**
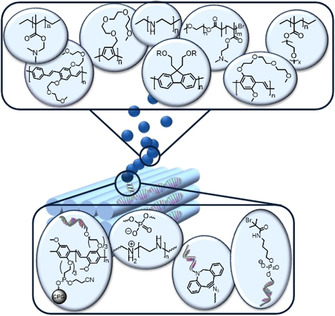
Schematic representation of polymer structures immobilized on DNA origami. The bottom box depicts the attachment mechanisms and moieties involved.

The combination of polymers and DNA origami has the power to merge the fields of synthetic and natural macromolecules, while getting the best of both worlds.[^19^, ^20^] On the one hand, the unprecedent addressability of DNA origami may organize polymers on the nanoscale into structures as polymers are typically known for entanglement and, thereby, pave the way for nanotechnological devices and structure‐function investigations. On the other hand, there is a large pool of polymers with a vast range of appealing and adjustable characteristics such as various charges, hydrophobicity or hydrophilicity, as well as stimulus‐responsiveness, and they may also stabilize DNA objects. There is a versatile range of ways to guide the process and achieve this fusion (Figure 2). Two fundamental strategies have to be distinguished here: Either the polymer is grown in situ on the DNA origami template (see Section 2.1) or the polymer is preformed and modified prior to conjugation to the DNA platform (see Section 2.2).

On the molecular level, the underlying principles for polymer attachment are manifold. The polymer can be electrostatically trapped to the negatively charged DNA backbone by the incorporation of respective positive counter charges or be bound to the DNA origami surface through base‐pair hybridization. The oligonucleotides required for this can be introduced by click reactions, by established bioconjugation techniques, or grown from nucleotides. Furthermore, hydrophobic interactions between the implemented polymers can be exploited to arrange polymer‐DNA constructs into higher ordered structures (see Section 2.3). However, the rather small number of publications in the field of DNA origami and polymer hybrids gives a first hint of how challenging this topic seems to be. Not only does synthesis suffer from various issues, such as solubility issues or steric hindrance of both the DNA and polymer reactive sites, but the characterization techniques are also very limited. One of the key drawbacks is the typically extremely low amount of DNA origami structures available, which impedes or even prohibits, for example, freeze‐pump‐thaw cycles for controlled radical polymerizations, sufficient amounts of attached initiator sites, or conventional polymer analysis by size‐exclusion chromatography (SEC), nuclear magnetic resonance (NMR), or dynamic light scattering (DLS).

### 2.1 Polymer Growth from DNA Origami

**Figure 3:**
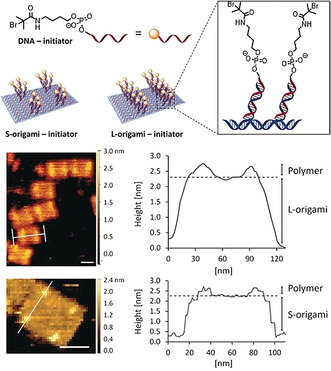
Top: Schematic representation of the DNA origami initiators used in the ATRP of PEGMEMA. Bottom: AFM images and height profiles of the L‐ and S‐origami after polymerization on DNA. Reprinted from Ref. [24] with permission.

The grafting from strategy is a convenient approach towards the synthesis of biomolecule‐polymer constructs with tailored properties, which are characterized by facile purification of the conjugate and commonly a high graft density.^[21]^ Controlled polymerization techniques have emerged as a powerful method to create polymers of controlled molecular weights and well‐defined architectures.^[22]^ Among others, atom transfer radical polymerization (ATRP) provides the possibility to conduct the polymerization under biologically relevant conditions that are suited to the stability of biomolecules, a low concentration of functional groups, or the presence of salts when working with buffers.^[23]^ However, successful polymerization from the biomolecule surface demands the installation of reactive handles which serve as initiator sites. We employed the highly precise scaffold of DNA origami to anchor ATRP initiators at predefined positions and, thereby, achieve directed polymer growth on the nanoscale (Figure 3).^[24]^ DNA origami sheets were equipped with different patterns of surface‐protruding, short oligonucleotide sequences. Complementary oligonucleotides were modified with ATRP initiators and attached to the DNA origami template by base‐pair hybridization. This macroinitiator was then utilized to induce the polymerization of poly(ethylene glycol) methyl ether methacrylate (PEGMEMA). This monomer was chosen because of its biocompatibility as well as its solubility in water, and the rather bulky side chains were considered to facilitate monitoring of the polymerization process by atomic force microscopy (AFM). Furthermore, the presence of sacrificial initiators (excess amount of free initiator DNA not attached to the DNA origami) was found to be crucial for successful polymer growth. Visualization of the origami structures by AFM, in particular recording the height profile, revealed the appearance of new objects where initiator sites were located at defined positions on the DNA nanotile. Furthermore, these objects have different mechanical properties which correspond to features of soft polymeric materials, such as PEGMEMA. Nevertheless, typical characterization of the polymer, such as determination of the chain length or dispersity by size‐exclusion chromatography, is not feasible here because of very low quantities. The incorporation of the bifunctional monomer PEG dimethacrylate (PEGDMA) to the polymerization process led to a cross‐linked polymer, whose structure could be preserved even after removal of the DNA template.

**Figure 4:**
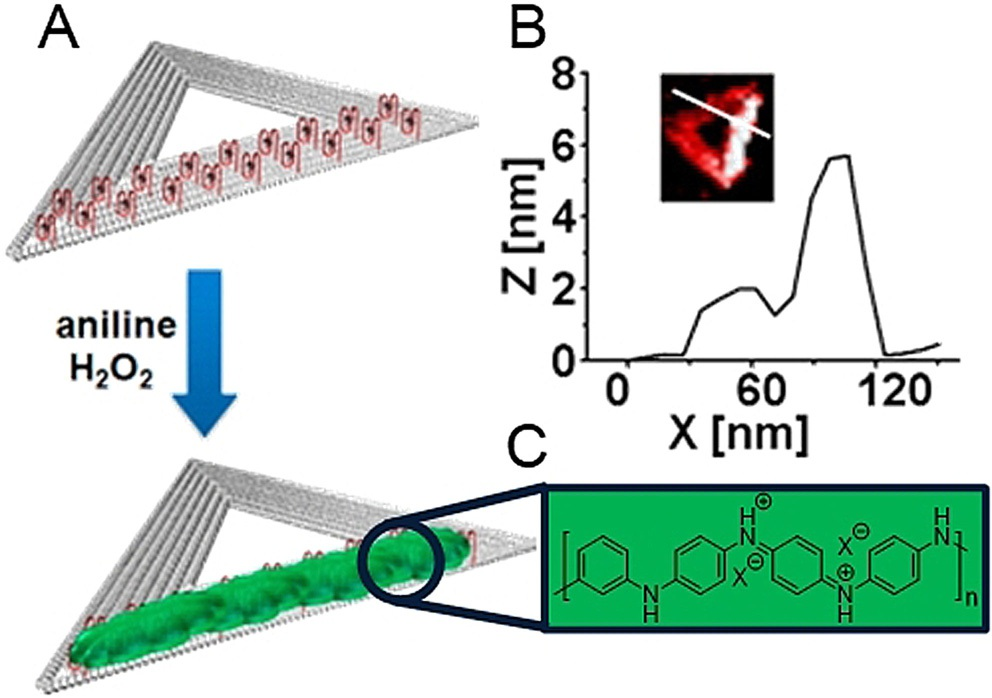
A) DNA‐templated synthesis of polyaniline (green) on origami triangles (gray) with DNAzymes (red structures with black dots) in the presence of aniline and H_2_O_2_. B) AFM image of a single PANI‐coated origami triangle. C) Structure of the emeraldine salt form of PANI. Reprinted from Ref. [26] with permission; Copyright (2020) American Chemical Society.

An essentially different polymerization technique, but also a grafting‐from strategy from DNA nanostructures, was introduced by Ding and co‐workers.^[25]^ They decorated a double‐stranded DNA template with guanine‐rich oligonucleotide sequences, the so‐called DNAzymes, which are capable of mimicking the activity of the enzyme horseradish peroxidase (HRP). Upon incorporation of the cofactor hemin and addition of hydrogen peroxide, the active DNAzyme catalyzes the polymerization of aniline. Thus, 1D polyaniline (PANI) structures were formed by a *para*‐coupling reaction, wherein the generated aniline radicals diffuse to the charged DNA surface. The regioselective formation of PANI was then transferred to 2D origami triangles (Figure 4).^[26]^ However, the use of DNA origami structures was challenging and required optimization of the reaction conditions: Whereas a high ionic strength disfavored the *para*‐coupling reaction of PANI, an insufficient Mg^2+^ concentration compromises the stability of the DNA folding. By AFM imaging, the group could show that polymer growth was favored around the DNAzymes and did not grow over the DNAzyme‐free regions. Thus, structural information transfer from the origami pattern to PANI was achieved, thereby leading to a polymer of predesigned geometry. Furthermore, the reversible redox behavior of polyaniline, which can be triggered by pH changes, renders these conductive hybrid objects promising candidates for the fields of electronics, sensors, and energy storage.

**Figure 5:**
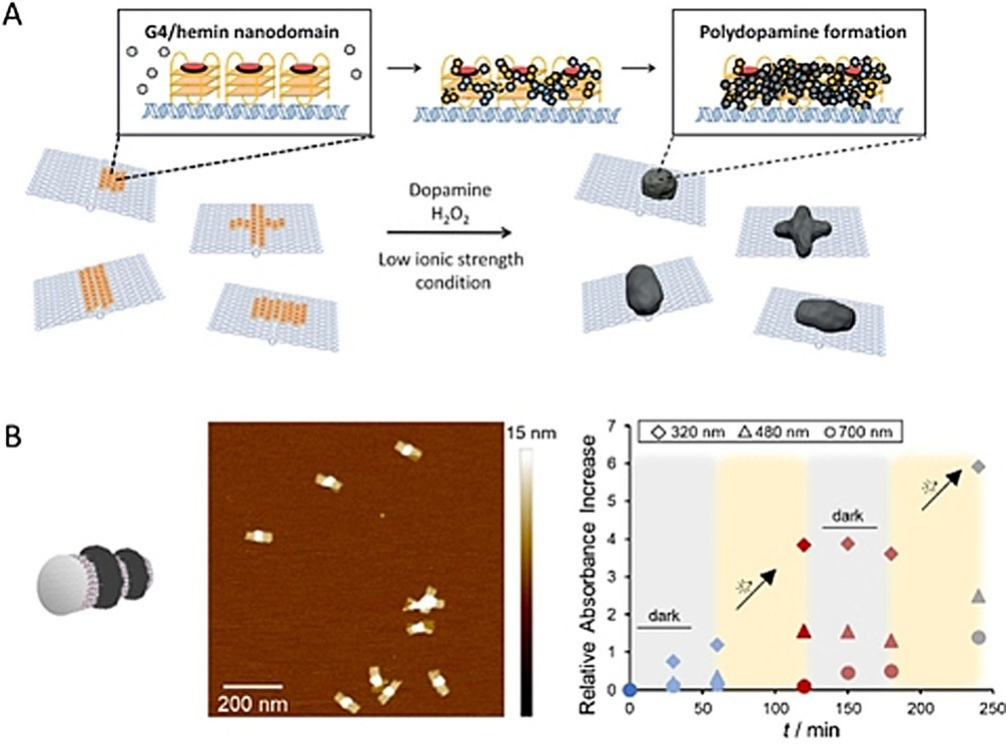
A) Chemically triggered formation of polydopamine on 2D DNA origami by DNAzymes to create highly precise hybrid objects.^[27]^ B) Photoinduced formation of polydopamine on 3D DNA origami under spatiotemporal control with the help of a locally trapped photosensitizer. Reprinted from Ref. [30] with permission.

The relatively simple and tolerant polymerization strategy was also applied to the polymerization of dopamine on DNA origami nanostructures.^[27]^ Polydopamine is a mussel‐inspired polymer which has aroused great interest among material scientists because of its excellent capability for surface functionalization.[^28^, ^29^] However, the self‐polymerization of dopamine and the not yet fully elucidated multifaceted polymer structure hamper its full potential. By employing the same DNAzymes as described above, we could induce and promote polydopamine formation on a 2D DNA nanosheet. It was essential to conduct the polymerization in an acidic milieu to suppress the self‐polymerization of dopamine and to gain control over the process. Different polydopamine shapes and sizes were obtained by arranging the catalytic centers in different patterns on the origami scaffold, and the reaction kinetics could be manipulated by altering the ionic strength and hydrogen peroxide concentration. The fabricated polydopamine nanostructures could serve as a “supramolecular glue”, thus guiding the origami conformation. This is an illustrative example of how the DNA template can affect the polymer formation and vice versa. In a follow‐up study, 3D origami structures were decorated with a photosensitizer, which was trapped at distinct positions by guanine‐rich oligonucleotides (G‐quadruplexes; Figure 5).^[30]^ Upon irradiation with visible light, dopamine was oxidized and polydopamine was deposited around the reaction centers. As a consequence of the light stimulus, the presence of hydrogen peroxide is no longer needed, which keeps the system simple. In addition, the polymerization process could be temporally controlled by simply switching the light on and off. In this way, photopatterned 3D nanostructures with dimensions far below 100 nm were created, which could not only preserve the DNA template in salt‐depleted media but they could also be released from the template under strong acidic conditions.

### 2.2 Polymer Attachment to DNA Origami

In all the examples discussed above, the polymer chain was grown from the DNA origami surface in distinct patterns, either covalently attached to the initiators by a controlled polymerization technique or noncovalently deposited next to the initiators on the DNA template. In contrast to this methodology, one can also make use of a preformed polymer, artificially synthesized or biologically derived, with graft‐suitable reactive handles and attach it to DNA nanostructures. Besides the rather intuitive idea of trapping a positively charged polymer by electrostatic interactions, it is also appealing to hybridize polymers by base‐pair recognition or to exploit the attractive area of click chemistry. However, many of the studies illustrate the boundaries of the bespoke strategies, such as the steric hindrance of polymers, their solubility, and the stability of DNA, which all impair successful conjugation.

#### 2.2.1 Electrostatic Interactions

The ionic nature of the phosphate backbone of DNA makes it possible to attach polymers through electrostatic interactions to DNA origami. Usually, the DNA origami structures are stabilized by the divalent cation Mg^2+^, which screens the negatively charged phosphate backbone of the DNA sequence to compensate charge repulsion between closely packed DNA strands. In a multitude of studies, the applied polymers comprise amino moieties in the side chain or backbone that undergo quaternization of the nitrogen atom when applied in acidic media. These polycations can then interact with DNA origami through ionic interactions.

**Figure 6:**
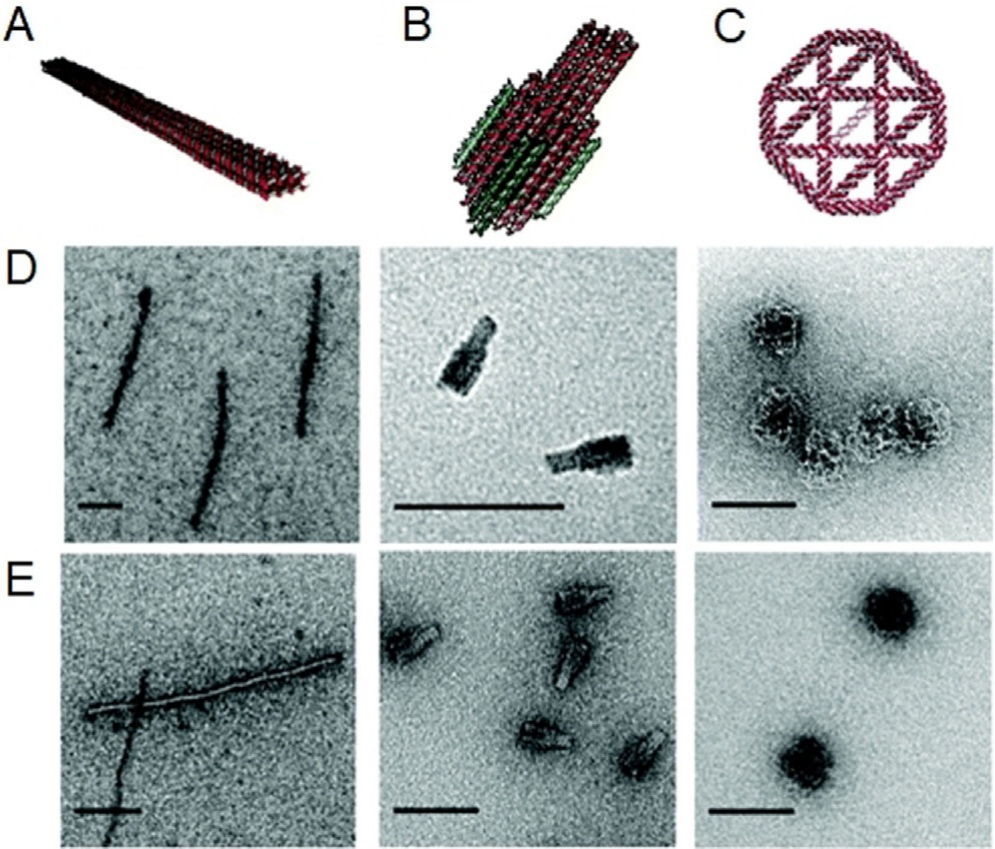
Representation of the different DNA origami structures. A) nanorod, B) nanobottle, and C) wireframe structure. D) TEM images of uncoated DNA origami structures. E) Origami structures stained with uranyl acetate after decomplexation with dextrane sulfate revealed intact origami structures. Reprinted from Ref. [32] with permission.

Based on this idea, Kiviaho et al. investigated the electrostatic binding between a 60‐helix‐bundled DNA nanostructure and cationic block copolymers.^[31]^ To assess the effect of the polymer structure on the binding affinity, the authors synthesized AB‐ and ABA‐type copolymers by ATRP. For this, they utilized a respective mono‐ and bifunctional PEG‐based macroinitiator to polymerize 2‐dimethylaminoethyl methacrylate (PDMAEMA), where the PEG moiety was intended to increase the poor biocompatibility of PDMAEMA. Coating was achieved by simply mixing the compounds under mild acidic conditions to ensure protonation of the amines. It could be demonstrated that all the polymers had a suitable binding efficiency but, interestingly, the block structure only had a minor impact. Instead, the ratio of total number of polymer amines and the total number of phosphates in DNA (referred to as the N/P ratio) was pivotal, irrespective of the arrangement of the nitrogen atoms in the polymer. Moreover, various polymer coatings were suited to control the activity of enzyme‐loaded DNA origami nanocontainers, as indicated by the bioluminescence reaction of luciferase enzymes. In a further study, commercially available linear polyethyleneimine (LPEI) and chitosan as a natural polymer were applied to form polyplexes with DNA origami nanostructures (Figure 6).^[32]^ The authors aimed to investigate several factors that might have an impact on the origami stability under physiological conditions, such as degree of polymerization, charge density, and nitrogen to phosphate ratio. Three different DNA objects were synthesized and applied for this purpose: a nanorod, a nanobottle, and a wireframe origami structure. After simple mixing of the DNA and polymer compounds, successful coating was demonstrated with the PicoGreen assay, which relies on the intercalation of the dye into the DNA double helix while exhibiting strong fluorescence. As a consequence of the polycation coating, PicoGreen was expelled from the polymer‐DNA complex, thereby resulting in a decrease in the fluorescence. Although bare origami could be imaged by negative‐stain transmission electron microscopy (nsTEM), the staining of the LPEI‐modified origami was only possible after removing the polymer coating by treatment with polyanionic dextran sulfate; this revealed intact origami structures and, thus, indirectly indicated successful encapsulation by the polymer. It could be shown that LPEI protects the structural integrity of the DNA origami more efficiently than chitosan and that this ability strongly depends on the nitrogen to phosphate (N/P) ratio. However, it must be considered that the unique addressability of the DNA origami surface might be masked by the polymer coating.

**Figure 7:**
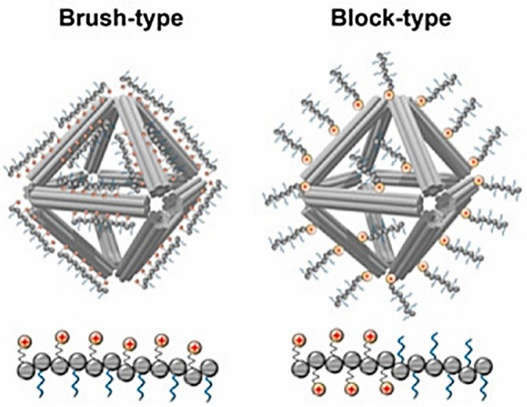
“Brush‐” and “block‐type” peptoids should lead to different surface coatings on octahedra‐shaped DNA origami. Reprinted from Ref. [35] with permission.

In 2017, two studies investigated the use of PEG‐oligolysine‐based copolymers to protect DNA origami structures against low‐salt denaturation and nuclease degradation, while the lysine block provides the positive charges to electrostatically interact with the DNA object, and the PEG is envisioned to have a shielding effect.[^33^, ^34^] The Schmidt group synthesized poly(ethylene glycol)‐*b*‐poly(l‐lysine) (PEG_12kDa_‐PLys_18_) by ring‐opening polymerization of *N* ^*ϵ*^‐trifluoroacetyl‐l‐lysine *N*‐carboxyanhydride initiated by an amine‐terminated 12 kDa PEG macroinitiator. In contrast to bare origami structures, polymer‐coated objects resisted the treatment of DNase I, fetal bovine serum (FBS), and low salt levels, and maintained structural integrity. However, the attachment of sterically demanding gold nanoparticles (AuNP) did not survive the process of polyplex formation; detachment could by visualized by transmission scanning electron microscopy (tSEM). The problem could be circumvented by employing shorter PEG chains, which still offer the same protection efficiency. These findings are in agreement with a similar study by Shih and co‐workers, who examined the beneficial contribution of the PEG_5kDa_PLys_10_ polymer coating to the origami stability. They could further prove that surface addressability of the DNA nanostructures was not constrained by the polymer film and immobilized ligands were capable of targeting receptors, thereby leading to cellular uptake of the hybrid objects. Very recently, Gang and co‐workers endeavored to also push the limits of the stability of DNA assembly in complex biological fluids (Figure 7).^[35]^ They put a novel class of polycationic polymers, namely peptoids, to the test. Peptoids are emerging peptidomimetics, whose side chains are not appended to the α‐carbon but to the nitrogen atom of the peptide backbone, thus, preventing secondary structure formation through hydrogen bonding and providing proteolysis resistance. In line with the approaches discussed above, the group explored the effect of peptoid architecture and sequence dependency on the origami stability. For this, they synthesized, by solid‐phase peptoid synthesis, brush‐ and block‐like peptoids that were built from positively charged monomers (electrostatic DNA complexation) and neutral oligo(ethyleneoxy) moieties (surface passivation). They could demonstrate that brush‐like peptoids were superior in protecting wireframed octahedra‐shaped DNA origami. Moreover, the capability of these structures to serve as a drug carrier with controlled release of doxorubicin was shown, which had not been achieved before. All these coating strategies are rather easily achieved by simply mixing the origami nanostructures with an excess of polymer, but they lack the possibility to arrange the polymer in distinct patterns.

#### 2.2.2 Oligonucleotide Hybridization

The attachment of polymers through ionic interactions, which are often just used to stabilize DNA, is very advantageous in terms of synthesis as well as the ease and straightforward fusion of a polymer and DNA origami. Nonetheless, this strategy does not consider the unique addressability provided by the DNA origami scaffold; on the contrary, it might even hinder it. Hence, the linkage of polymers to DNA objects by complementary base‐pair recognition allows the highly precise positioning of single polymer chains and overcomes their lack of intrinsic self‐assembly properties. To equip polymers with the necessary handles, namely, oligonucleotides that are complementary to ssDNA sequences on the origami surface, one can either functionalize the polymer's end group or the side chains accordingly. It has been proven useful to either “click” the oligonucleotide to the polymer or to grow oligonucleotides directly from the polymer backbone. In both cases, hybridizing the respective polymer to the DNA origami is always reversible and should permit programmed switching.

Gothelf and co‐workers see a great prospective in binding conjugated and, therefore, potentially conducting polymers on DNA origami templates to build molecular‐scale electronic or optical wires.^[36]^ For this purpose, they synthesized a conjugated poly(phenylenevinylene) polymer with alkoxy side chains (APPV) from a dithiocarbamate precursor. Each phenylene unit in the backbone bears a triethylene glycol side chain and with the help of protective group chemistry, a small number of hydroxy groups were employed to attach the polymer to the solid support; the remaining hydroxy groups were used in automated solid‐phase DNA synthesis to graft 9‐mer oligonucleotides. By this approach, they obtained a fully water‐soluble brush polymer with ssDNA extending from the majority of the repeating units. However, the size distribution was rather broad, as characterized by gel‐permeation chromatography (GPC; 340–3300 kDa) and AFM (lengths in the range of 20 nm to 200 nm), which the authors explain through partial degradation during purification. By equipping 2D and 3D DNA origami templates with complementary oligonucleotide sequences, they could link single polymer chains to the template in different geometries. Moreover, they could observe Förster resonance energy transfer (FRET) between the attached polymer (donor) and a co‐immobilized acceptor dye, thereby proving that absorption and emission of the polymer backbone is not harmed by the applied methods. Further studies that exploit this strategy towards the development of nanocircuits are discussed in Section 3.

**Figure 8:**
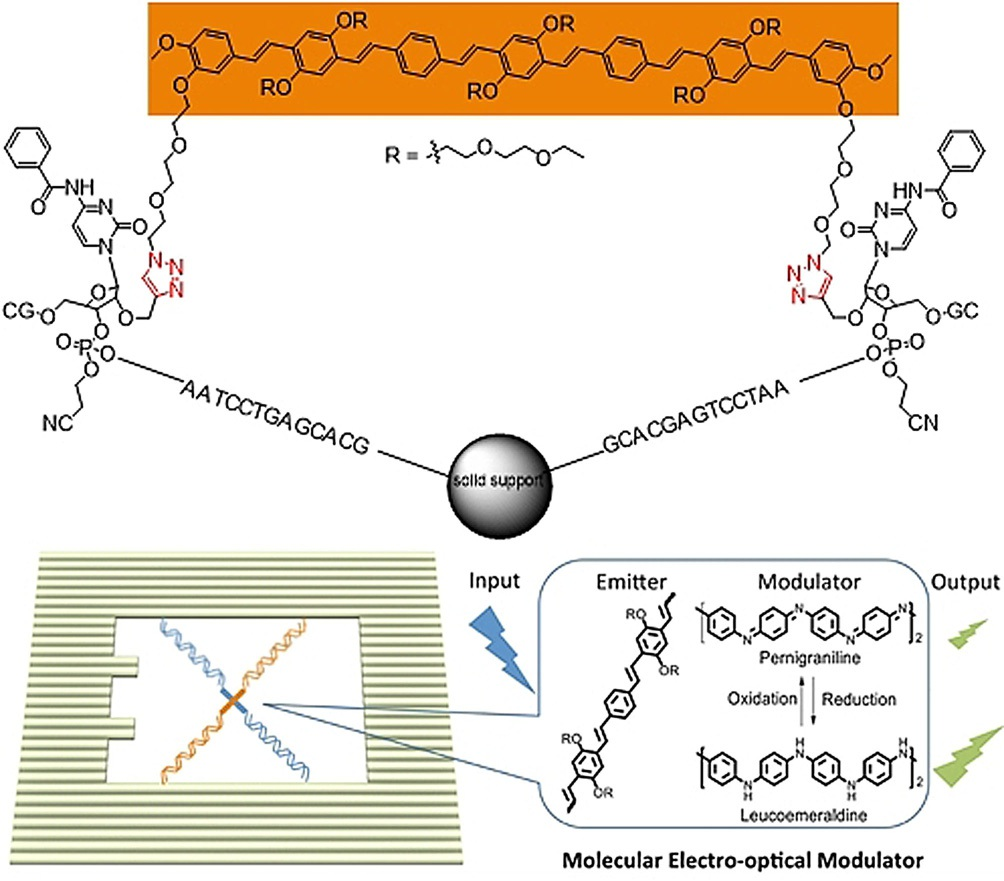
Top: Product of the double‐CuAAC reaction between an azide‐functionalized oligomer (OPV) and an alkyne‐functionalized oligonucleotide sequence, immobilized on a solid support (controlled pore glass). Bottom: Schematic representation of the x‐shaped OANI‐ and OPV‐DNA conjugate on the DNA origami frame. OANI (blue) acts as a modulator of the fluorescence intensity of the OPV (orange). Reprinted from Ref. [37] with permission. Copyright (2020) American Chemical Society.

In contrast to the rather sophisticated and challenging solid‐phase synthesis of oligonucleotides directly from the polymer backbone, one can also furnish the polymer with a suitable end group and “click” it to the respective ssDNA. In this context, copper‐catalyzed azide–alkyne reactions (CuAAC)^[37]^ as well as a copper‐free variant involving a strain‐promoted azide–alkyne click reaction (spAAC)[^38^, ^39^] have been utilized in different studies. For the development of a DNA origami assisted electrooptical modulator, Canary and co‐workers equipped two different kinds of organic semiconductors, namely oligomers of poly(phenylene vinylene) (HPV) and oligoaniline (OANI), with ssDNA strands, which allows their attachment to a DNA origami scaffold (Figure 8).^[37]^

**Figure 9:**
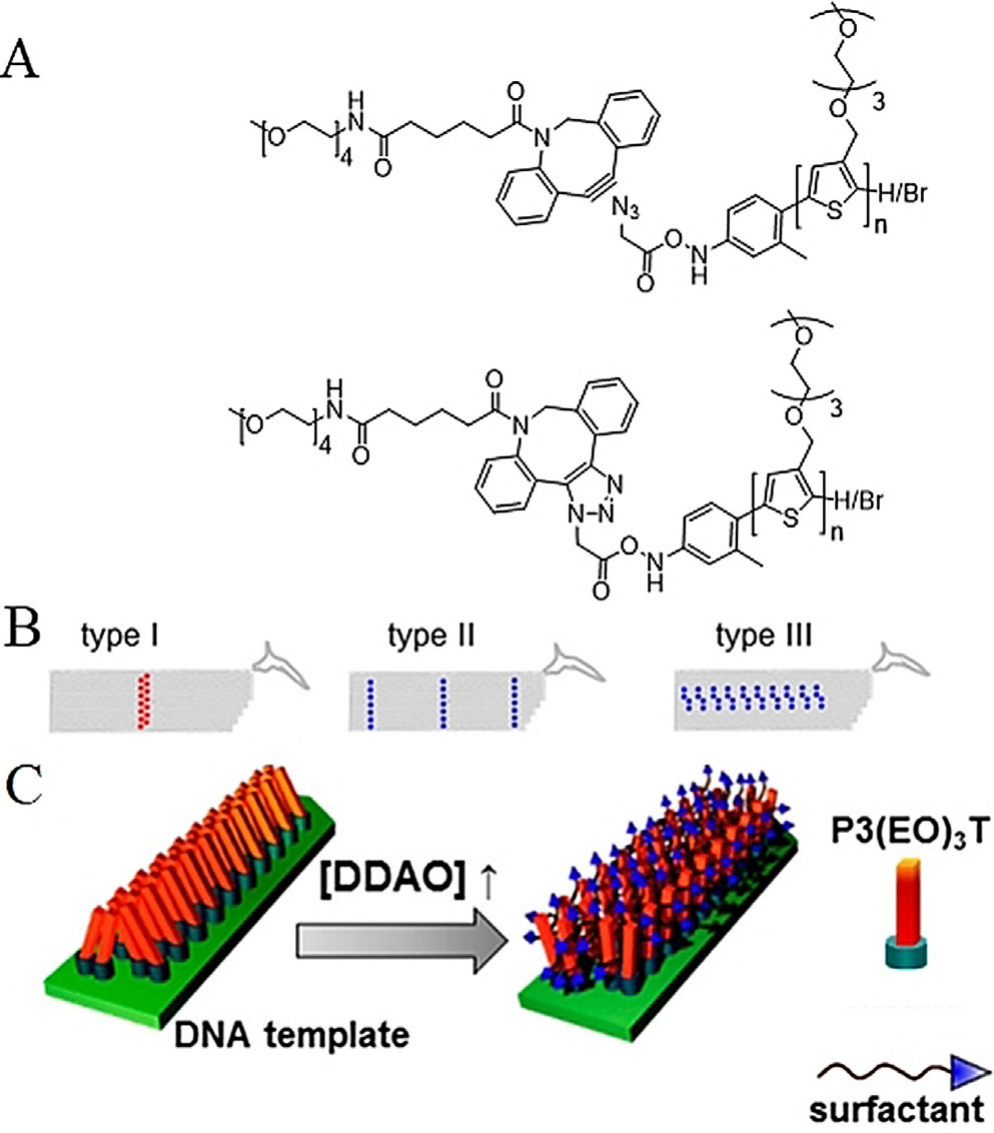
A) spAAC reaction with a cyclooctyne‐functionalized oligonucleotide and azide‐functionalized polythiophene. B) The three different DNA origami pad types. C) Illustration of aggregated P3(EO)_3_T on the DNA origami (left) and the deaggregated structure (right) after the addition of surfactant. Printed from Ref. [38] with permission. Copyright (2020) American Chemical Society.

By this approach, the symmetric oligomers with azide groups at each end were “double‐clicked” by CuAAc to oligonucleotide strands containing a propargyl residue. Consequently, the obtained structure consisted of a polymer with oligonucleotide sequences at both ends. By hybridizing the polymer‐DNA constructs to a DNA origami frame with four complementary anchor strands, the semiconductors were brought into proximity, thereby forming a cross‐like structure. By tuning the oxidation state of polyaniline, the energy transfer from HPV to OANI could be tuned, as visualized by an altered fluorescence signal. However, the hybridization efficiency of only 20 % correctly formed polymer‐DNA origami structures (determined by AFM) illustrates how challenging the formation of these hybrid objects is, although base‐pair hybridization is often assumed to be straightforward. Mertig and co‐workers also employed click reactions to conjugate conducting polymers in distinct patterns to an origami surface (Figure 9).^[38]^ For this, they synthesized well‐defined thiophene‐based polymers with dispersities between 1.1 to 1.3 by Kumada polycondensation. Oligoethylene glycol bearing side chains ensured water solubility of the polymer and thereby allowed reaction of the azide‐functionalized polymer and dibenzocyclooctyne‐end‐capped oligonucleotide in aqueous solution. It is noteworthy that the degree of functionalization of the polymers is only in the moderate range of 38–71 %. However, unfunctionalized polymer chains are not considered to participate in, or even harm, further transformations. Three different oligonucleotide sequences were conjugated to the polymers and were attached to three different DNA origami pads with patterns of respective handles to study sequence‐hybridization effects. Virtually all the pads displayed at least one attached object, but the overall occupation probability per handle was roughly one third. For example, 4 out of 14 handles on one origami pad displayed an attached object. This again indicates that hybridization of polymers to DNA surfaces is difficult and sterically demanding. Applying surfactants to polymer‐decorated origami was accompanied by a blue‐shifted increase in the fluorescence and, thus, indirectly showed that interchain π‐π stacking of polythiophene units occurs. This feature might offer the possibility to fine‐tune optical properties on a molecular level.

### 2.3 Higher Order Structures

In addition to the attachment methods discussed above, one can also make use of the hydrophobic effect to form higher ordered structures built from DNA origami and polymers. By applying hydrophobic polymers to DNA scaffolds, surface properties can be altered significantly and, thus, self‐assembly of amphiphilic structures can be induced.

**Figure 10:**
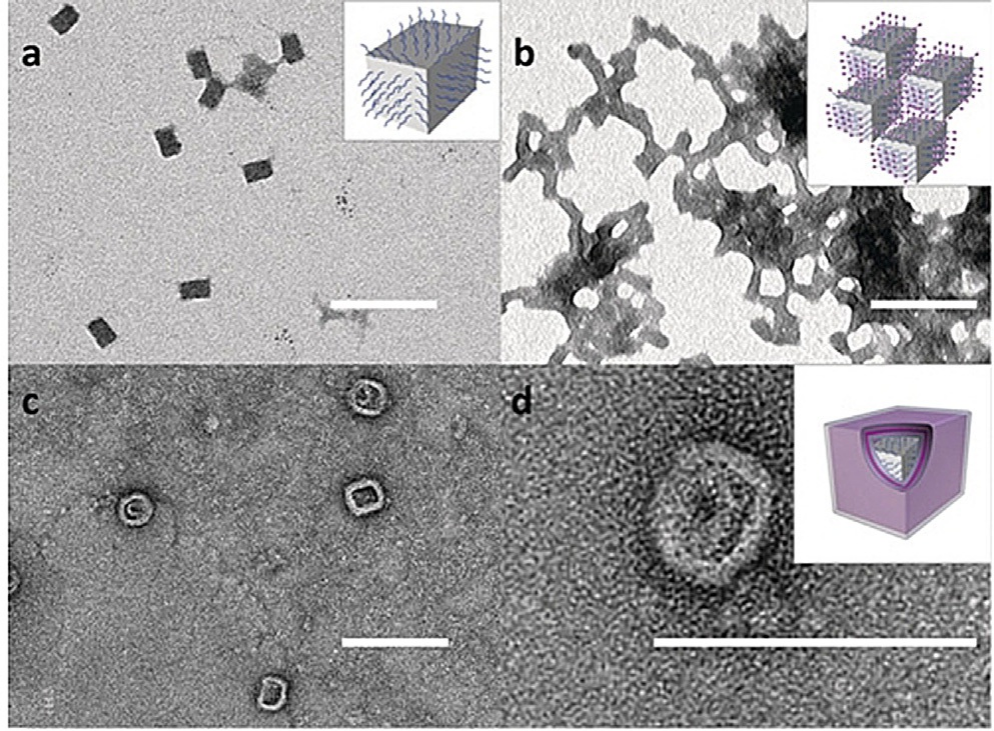
TEM images of the DNA‐cube‐dendron aggregates. a) Origami cuboid. b) Aggregates formed upon addition of D_T_DOEG. c,d) Heterovesicles formed from the aggregate after the addition of G_2_Cl 18. Reprinted from Ref. [40] with permission.

In 2015, Liu and co‐workers showed that attaching hydrophobic dendrons to DNA origami rectangles could lead to the formation of surface areas with a high local concentration of hydrophobic molecules, which, as a result of the hydrophobic effect, guided origami folding into various thermodynamically stable products. Poly(aryl ether) dendrons were conjugated to oligonucleotides through solid‐phase synthesis, whereas modification with oligo(ethylene glycol) tails should increase the water solubility of the dendrons. Upon traditional origami annealing in the presence of both elongated capture strands (handles on the origami) and oligonucleotide‐bearing dendrons (complementary to handles on the origami), sandwich‐like structures were created. In a follow‐up study, the same group created polymer vesicles on the shell of a DNA origami cube (Figure 10).^[40]^

The attachment of the above‐mentioned hydrophobic dendrons to origami cubes led to aggregation and precipitation events, most likely because of π‐π stacking between several cuboid frames (frame–frame interactions). The addition of a second hydrophobic dendron, the so‐called principal amphiphile (PA), to the amphiphilic construct breaks the frame–frame interactions and promotes stronger PA–frame interactions, thereby resulting in the formation of heterovesicles. To demonstrate the applicability of this process to different molecules, the dendrons were substituted by polymers: DNA cuboids were covered with thermoresponsive poly(propylene oxide) (PPO) and upon heating, the polymer became hydrophobic and, thus, guided a second PPO polymer to form heterovesicles.

This is an impressive example of higher order assemblies based on polymer‐decorated DNA origami structures; however, the intricate technique requires a strong background in the field of frame‐guided assembly to be successful. The use of hydrophobic interactions between polymers attached to 3D DNA nanostructures was also shown to yield larger DNA micelles.[^41^, ^42^] For this purpose, DNA nanostructures of three different forms (trigonal prism, cube, and pentagonal prism) were decorated with oligonucleotides covalently linked to hexaethylene phosphate to yield DNA nanostructures with polymer strands.^[41]^ It could be revealed that the number of hexaethylene phosphate repeating units is crucial for the micellization: At least six of these repeating units are required to form higher ordered structures, with micellar structures being observed when the number of repeating units is increased to at least eight.^[41]^ Not only were micelles with cubic DNA structures synthesized, but trigonal and pentagonal prisms were also obtained. TEM, AFM and DLS were utilized to compare the micelles of the different DNA structures, which revealed that they appear to have approximately the same size and that the size is only influenced by the number of repeating units of the hexaethylene phosphate. The group further investigated the influence of a combination of hydrophobic and hydrophilic repeating units in the DNA polymer conjugates attached to the prismatic structures.^[42]^ The hydrophobic block consisted of 1,12‐dodecanediol (HE), and the hydrophilic block was represented by hexaethyloxy glycol (HEG). First experiments combined the cubic DNA structure with four DNA copolymer strands, which consisted of six HE and six HEG units, in different orders. Notably, higher mobility in the gel electrophoresis was observed as the HE block length was increased, which was explained by the folding of the polymer chains into the cage structure.^[42]^ The nanostructure with the HE_6_HEG_6_ block copolymer was exceptional, as it formed rings of three to five polymer‐decorated DNA cubes in a doughnut‐like fashion instead of the expected micellar structures. By increasing the length of the hydrophilic HEG block, the diameter of the ringlike assemblies could be increased, in contrast to the HE_6_HEG_6_ block copolymer, which indicates that the HEG block acts as a spacer.^[42]^ Remarkably, the formation of micelles was not observed when (block) copolymers consisting of hydrophobic and hydrophilic units were used. These can only be observed in the case of hydrophobic polymers.^[42]^ The previously discussed examples show that not only can DNA origami be utilized to direct polymers into larger structures and desired shapes, but polymer–polymer interactions also allow macromolecular structures to be created with prior‐folded DNA origami.

## 3 Next‐Generation Polymeric Hybrid Materials: Fields of Application

In the previous section, we highlighted different techniques for linking polymers and DNA origami as well as the influence on each other. Although there are fewer examples than one might expect regarding the potential provided by these materials, and although there are still some challenges to overcome, several studies report the first steps towards future applications and prospects.

**Figure 11:**
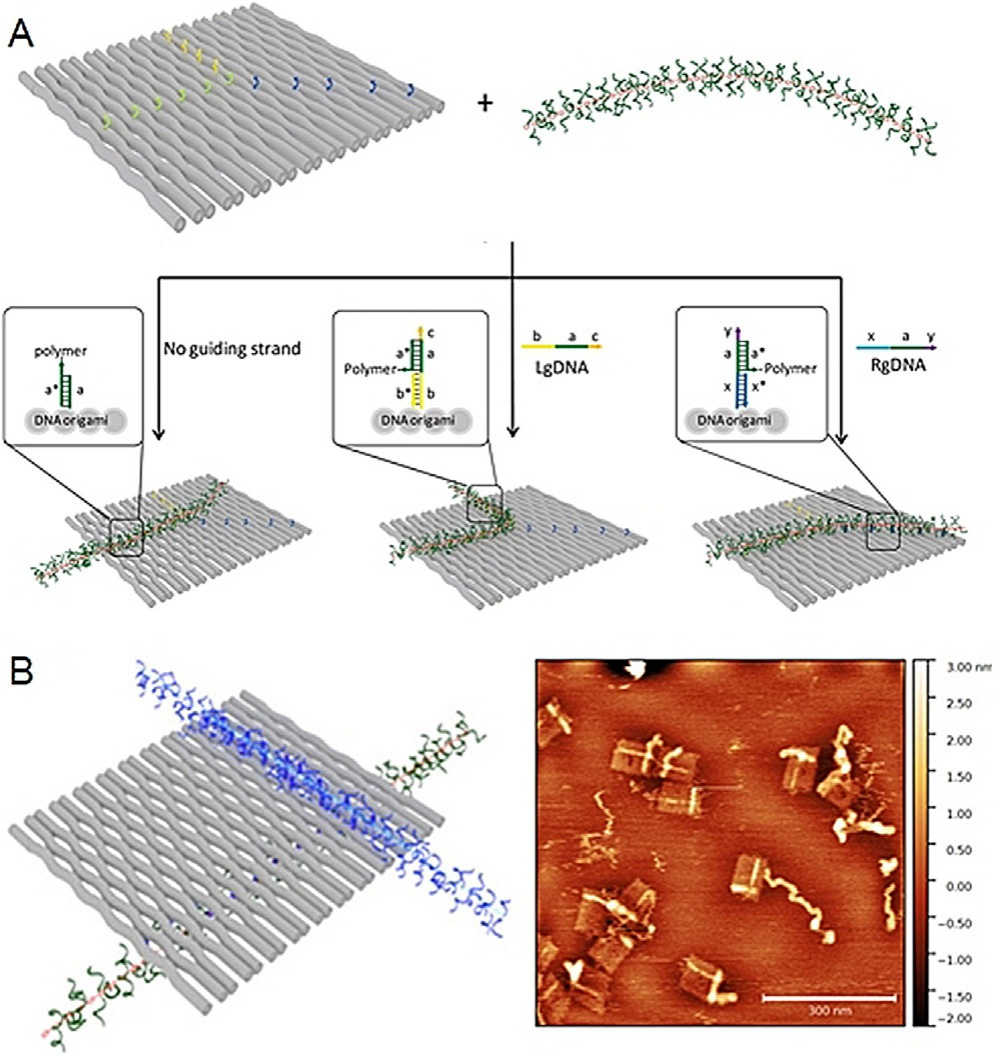
A) Switching of a polymer strand conformation on a DNA origami by adding guiding and remover strands. B) p(F‐DNA) (blue) and p(PPV‐DNA) (green) on a DNA origami tile with the AFM image (right). Reprinted from Refs. [43, 44] with permission. Copyright (2020) American Chemical Society.

Gothelf and co‐workers exploited their system of attaching a conjugated brush‐like polymer with oligonucleotide side chains onto DNA origami tiles to contribute to the area of nanophotonic and nanoelectronic devices. They not only attached the polymer to the origami platform, they also precisely forced the polymer to switch its position and conformation (Figure 11 a).^[43]^ For this, two sets of so‐called guiding strands were employed that allow the polymer to follow two different routes on the origami tile, depending on which type of guiding strand is applied. The guiding strands are also equipped with a toehold region—a short sequence of nucleotides which does not take part in polymer hybridization. Hence, the guiding strands can be trapped by a fully complementary remover strand, which leads to the release of the polymer. By subsequently adding the other set of guiding strands, the polymer can be routed along the second track on the origami. These events can be monitored by FRET between the polymer and arranged reporter dyes. It should be noted that approximately only half the origami structures displayed well‐aligned polymers (AFM) and that surface contamination after the conformation switch significantly harmed imaging. In an ensuing study, the group aimed to investigate the interaction between two different types of conjugated polymers by making use of the unique addressability of DNA origami (Figure 11 b).^[44]^ In addition to the above‐mentioned APPV‐DNA copolymer, they similarly synthesized a polyfluorene‐DNA pendant (poly(F‐DNA)). However, no interpolymer energy transfer was observed on conjugating either polymer to the origami rectangle. This might be caused by a lack of interpolymer contact in combination with interference from unbound polymers, which demonstrates the limits for the conjugation of intricate polymers. The polymers were directly co‐localized by hybridization of the side chains for further investigations.

**Figure 12:**
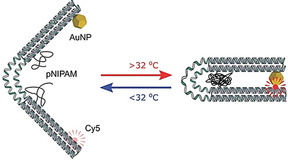
Representation of the DNA origami flexor. Upon heating of the flexor‐containing solution, PNIPAM aggregates and leads to a change in the DNA origami shape. Reprinted from Ref. [39] with permission.

DNA origami is an emerging platform to direct the motion of various objects on the nanoscale; however, the movement of the attached objects is often “fuel‐based”, that is, employing strand displacement reactions of respectively designed oligonucleotides to break and create old and new bonds. Thus, purification after each step is often necessary. Baumberg and co‐workers developed a DNA origami flexor whose actuation is mediated by a thermoresponsive polymer which can be stimulated externally (Figure 12).^[39]^

They designed a flexible DNA origami hinge structure, whereby poly(*N*‐isopropylacrylamide) (PNIPAM) was attached on either side of the hinge. In this way, a PNIPAM‐DNA conjugate was formed by catalyst‐free strain‐promoted cycloaddition and attached to the complementary capture strands within the hinge region. By fixing a gold nanoparticle and a fluorescent dye at opposite ends, the switch between the opened and closed state of the hinge could be optically monitored. Upon heating above the lower critical solution temperature (LCST) of 32 °C, PNIMPM becomes hydrophobic and forces the hinge to close. This could be conclusively tracked by an increase in fluorescence as well as changes in the size distribution (DLS). However, the AFM images obtained are a vivid example of how difficult direct visualization of conformation‐altering DNA origami structures can be.

**Figure 13:**
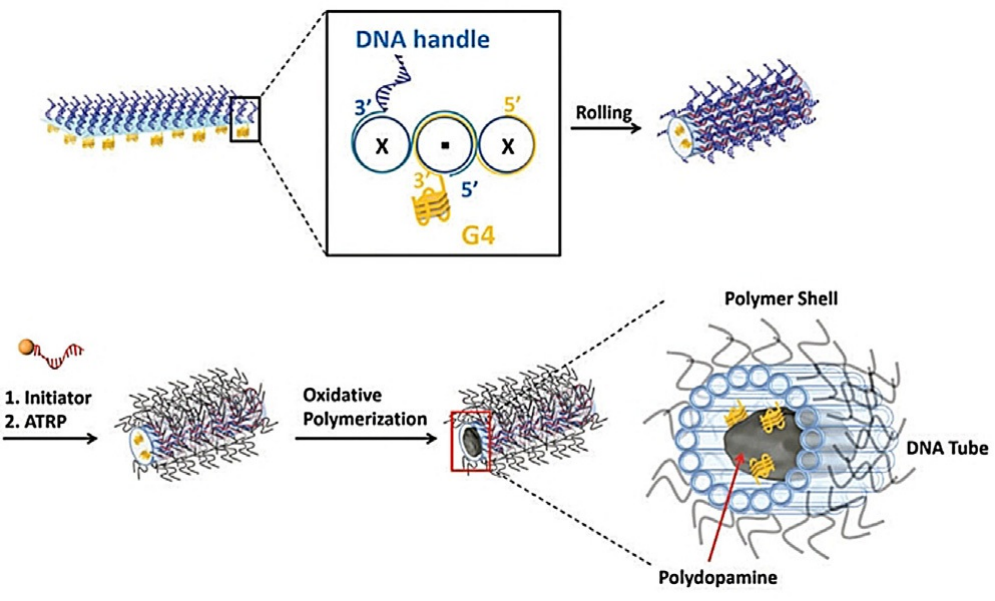
Folding of the DNA origami tube and decoration with oligonucleotide‐modified ATRP initiators and the subsequent polymerization of PEGMEMA and PEGDMA. Reprinted from Ref. [45] with permission (published by The Royal Society of Chemistry).

Tokura et al. further developed their surface‐initiated ATRP on a DNA origami tile by transferring the technique to a 3D tube, preliminary paving the way towards 3D engineering of nanomaterials (Figure 13).^[45]^ The authors designed a system where orthogonal polymer growth is feasible: After coating of the outer surface with cross‐linked PEGMEMA, the inner cavity of the origami tube was equipped with DNAzymes to induce the polymerization of dopamine. Whereas AFM images captured after the first polymerization step could reveal an increase in the height profile and, thus, the presence of polymer, no imaging was possible of the polymer synthesized in the inside. The formation of polydopamine could only by monitored by absorbance spectroscopy, once more emphasizing how complicated the characterization of polymer‐DNA hybrid objects is.

## 4 Summary and Outlook

The combination of DNA origami and polymers is a strong and emerging tool towards precise surface modification and the creation of elusively defined nanostructures in the low nanometer regime, thus, representing a kind of a “top‐up” approach that merges conventional bottom‐up and top‐down techniques. To date, the arrangement of polymeric objects in a virtually infinite variety of geometries with precision of a few nanometers is not reported by any other methodology. It thereby pushes the limits of established lithography and self‐assembly approaches by programming distinct nanodevices. Furthermore, DNA origami allows orthogonal decoration of polymers and other molecules, thereby enabling the investigation of energy‐transfer processes, as well as the installation of suitable reporter systems or targeting groups. In principal, two different strategies lead to the formation of such hybrid structures: either the polymer is grown from the DNA origami template or a preformed polymer is linked to the DNA platform. With regard to the studies discussed herein, it turns out that there are significantly more reports within the latter category. The grafting of polymers from the origami surface is very challenging due to the extremely low concentration of DNA objects and the, therefore, small number of initiator sites as well as the increased sensitivity to oxygen present because of the ultralow reaction volumes. Moreover, it is not possible to determine average molecular weights and distributions of the grown chains. Furthermore, the attachment of polymers to DNA origami faces some hurdles: the solubility of the polymer is preferred to be compatible with DNA, and the entanglement of polymers and the folding of DNA might shield their reactive centers. However, this strategy allows the larger scale synthesis of polymers and their thorough characterization prior to DNA origami fusion.

Whereas the electrostatic coating of DNA nanostructures with polycations may be considered as straightforward, it often only aims to stabilize the inherently susceptible DNA construct in biologically relevant media, but does not exploit the addressability of the platform to achieve molecular patterning. Therefore, hybridization of respectively modified polymers to complementary capture strands on the DNA origami is more expedient, but the conjugation efficiency and the grafting density is often reported as rather low. We regard it as important to once again emphasize the characterization challenges which come along with the synthesis of polymer‐DNA origami hybrid structures and which hinder fast progress in the field. The ultrasmall quantities of DNA origami hamper typical polymer analysis methods such as SEC, NMR, or DLS. To monitor the impact of polymers on DNA origami at a qualitative level, agarose gel electrophoresis can be employed. However, the integrity of the structures cannot be confirmed in this way. Therefore, imaging techniques such as AFM and TEM have to be performed to visualize the objects. As a consequence of the small size of DNA origami, such techniques have to be operated in high‐resolution modii, and sample preparation, for example, drying effects, has to be taken into account. The most representative image might be captured by performing AFM in a liquid environment, which corresponds to the natural occurrence of DNA origami in aqueous solution. Thus, indirect characterization, for example, FRET, can also be utilized to monitor conformation changes.

In conclusion, the fusion of polymers with DNA origami holds great potential for designing programmable nanodevices with highest structural precision, and there are already pioneering investigations towards the application of this class of new materials.

## Conflict of interest

The authors declare no conflict of interest.

## Biographical Information

*Nadine Hannewald studied chemistry at the Friedrich Schiller University in Jena and graduated in 2018 in the field of organic and macromolecular Chemistry. She is currently working as a PhD student in the group of Prof. Schubert, where she focuses on the synthesis of well‐defined polymer architectures for attachment to DNA origami*.

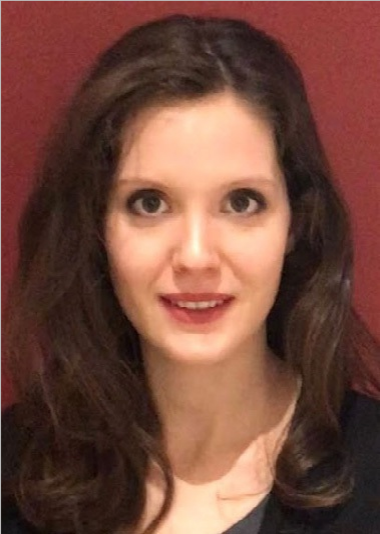

## Biographical Information

*Pia Winterwerber studied Biomedical Chemistry at the Johannes Gutenberg University, Mainz (Germany), and received her master's degree in 2018. During her studies she focused on the synthesis of hyperbranched star polymers as well as amphiphilic biomacromolecule conjugates. She joined the group of Prof. Dr. Tanja Weil at the Max Planck Institute for Polymer Research, Mainz, in 2018 as a PhD student, and is now working on the development of DNA origami hybrid nanostructures*.

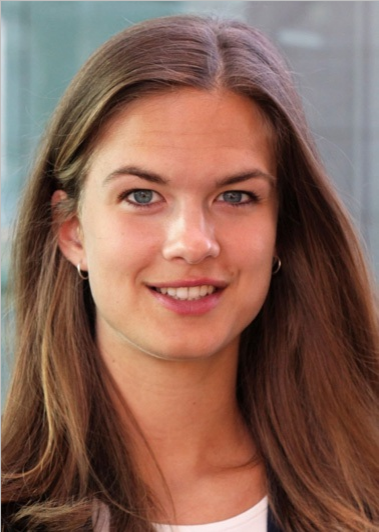

## Biographical Information

*Tanja Weil studied chemistry at the TU Braunschweig (Germany) and the University of Bordeaux (France) and completed her PhD at the Max Planck Institute for Polymer Research (MPIP) in Mainz, for which was awarded the Otto‐Hahn Medal in 2003. After several years in industry, she returned to academia by accepting an Associate Professor position at the National University of Singapore in 2008. In 2010, she joined the University of Ulm as Director of the Institute of Organic Chemistry III. Since 2017, she has been Director at the MPIP, heading the department “Synthesis of Macromolecules”*.

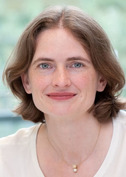

## Biographical Information

*Ulrich S. Schubert studied chemistry in Frankfurt and Bayreuth (both Germany) and at Virginia Commonwealth University, Richmond (USA). After PhD studies at the Universities of Bayreuth and South Florida, and postdoctoral training with J.‐M. Lehn, he moved to the TU Munich (Germany), where he obtained his Habilitation in 1999. 1999–2000 he was Professor at the University of Munich and 2000–2007 Full Professor at the TU Eindhoven (The Netherlands). Since 2007, he has been a Full Professor at the Friedrich Schiller University Jena (Germany). He is an elected member of the German National Academy of Science and Engineering (acatech) and external scientific member of the Max‐Planck‐Gesellschaft (MPI for Colloid & Interfaces, Golm)*.